# Promoter Methylation of the Retinoic Acid Receptor Beta2 (RARβ2) Is Associated with Increased Risk of Breast Cancer: A PRISMA Compliant Meta-Analysis

**DOI:** 10.1371/journal.pone.0140329

**Published:** 2015-10-09

**Authors:** Cheng Fang, Zhi-Yuan Jian, Xian-Feng Shen, Xue-Mei Wei, Guo-Zheng Yu, Xian-Tao Zeng

**Affiliations:** 1 Center for Evidence-Based Medicine and Translational Medicine, Zhongnan Hospital of Wuhan University, Wuhan, China; 2 Department of General Surgery, Taihe Hospital, Hubei University of Medicine, Shiyan, China; 3 Department of Nursing, Affiliated Hospital of North Sichuan Medical College, Nanchong, 637000, P.R. China; University of North Carolina School of Medicine, UNITED STATES

## Abstract

**Background:**

Epigenetic studies demonstrate that an association may exist between methylation of the retinoic acid receptor beta2 (RARβ2) gene promoter and breast cancer onset risk, tumor stage, and histological grade, however the results of these studies are not consistent. Hence, we performed this meta-analysis to ascertain a more comprehensive and accurate association.

**Materials and Methods:**

Relevant studies were retrieved from the PubMed, Embase and Chinese National Knowledge Infrastructure databases up to February 28, 2015. After two independent reviewers screened the studies and extracted the necessary data, meta-analysis was performed using Review Manager 5.2 software.

**Results:**

Nineteen eligible articles, including 20 studies, were included in our analysis. Compared to non-cancerous controls, the frequency of RARβ2 methylation was 7.27 times higher in patients with breast cancer (odds ratio (OR) = 7.27, 95% confidence interval (CI) = 3.01–17.52). Compared to late-stage RARβ2 methylated patients, the pooled OR of early-stage ones was 0.81 (OR = 0.81, 95% CI = 0.55–1.17). The OR of low-grade RARβ2 methylated patients was 0.96 (OR = 0.96, 95% CI = 0.74–1.25) compared to high-grade RARβ2 methylated patients.

**Conclusion:**

RARβ2 methylation is significantly increased in breast cancer samples when compared to non-cancerous controls. RARβ2 could serve as a potential epigenetic marker for breast cancer detection and management.

## Introduction

Breast cancer is the most frequently diagnosed malignancy and the leading cause of death in women, accounting for 23% of all cancer deaths worldwide [1]. The incidence rate of this disease has been increasing 3% annually in Asian countries [2]. Approximately 232,340 new cases of invasive breast cancer were diagnosed and about 39,620 cancer deaths occurred among women in the United States in 2013 [3]. Despite advances in early detection through mammography screening, hurdles in the early diagnosis and treatment of breast cancer still exist [4]. Thus, novel approaches in the diagnosis and prevention of this disease merit investigation.

Aberrant methylation of CpG islands within the promoters and 5’-end regulatory regions of genes is increasingly being recognized as a frequent epigenetic modification and has been associated with transcriptional silencing of gene expression in mammalian cells [5]. Recent studies have demonstrated that epigenetic changes of cancer-related genes due to the methylation of gene promoter regions are early events in human carcinogenesis [6–7]. The human retinoic acid receptor beta2 (RARβ2) is a member of the nuclear receptor super-family and plays a key role in modulating the effects of retinoic acid (RA) on cell growth and differentiation [8]. RARβ2 is an isoform of the RARβ gene transcribed by the P2 promoter located at 3p24 [9]. Importantly, RARβ2 may act as an effective inhibitor of oncogene-induced focus formation, similar to the tumor suppressor gene p53 [10]. In addition, down-regulation of RARβ2 mRNA expression has been observed in numerous malignant cell lines, including breast carcinoma [11–14]. In these cases, DNA methylation has been found to be responsible for the observed decrease in transcription of RARβ2 [13–14]. Furthermore, hypermethylation of the RARβ2 promoter is frequently reported to occur in breast cancer [14–16]. Aberrant promoter methylation of RARβ2 suppresses the expression and function of the RARβ2 transcript, leading to dysregulation of the cell cycle, thus promoting mammary carcinogenesis. These findings suggest the potential utility of RARβ2 as a molecular predictor of tumor progression.

In recent decades, methylation patterns of the RARβ2 gene promoter have been extensively studied in both tissue and blood samples of breast cancer patients. However, the functional significance of RARβ2 promoter methylation in the diagnosis of breast cancer, and the association between RARβ2 methylation and breast cancer stage or histological grade still need to be determined. Therefore, this meta-analysis was conducted to achieve a more accurate assessment of the role of RARβ2 promoter methylation in breast cancer pathogenesis and development.

## Methods

### Protocol register

This meta-analysis was reported according to the Preferred Reporting Items for Systematic Reviews and Meta-Analysis (PRISMA) statement [17] (S1 PRISMA Checklist). The protocol of this meta-analysis was registered in PROSPERO (http://www.crd.york.ac.uk/prospero/), and the registration number is CRD42014015688.

### Eligibility criteria

Studies were considered applicable if they met all the following criteria: (1) the study design was a case-control or case-series; (2) investigated the correlation between RARβ2 promoter methylation and breast cancer; (3) provided sufficient information about the frequency of RARβ2 promoter methylation in tissue or blood samples of cancer patients; (4) the numbers of patients and controls were no less than five; (5) RARβ2 methylation was examined by methylation-specific polymerase chain reaction (MSP) or quantitative MSP (QMSP). Additionally, when overlapping data of the same patient population were reported in more than one publication, only the most recent or complete study was used in this analysis. All eligible articles were carefully identified in duplicate by two independent investigators.

### Search Strategy

The PubMed, Embase and Chinese National Knowledge Infrastructure (CNKI) databases were searched for relevant studies up to February 28, 2015 using the following keywords: (breast cancer OR mammary cancer) AND (RARβ2 OR retinoic acid receptor beta2 OR RARbeta2) AND (methylation OR hypermethylation). A manual search of the references of included articles and recent reviews was also conducted.

### Data extraction

The information extracted from each eligible study were as follows: first author’s name, publication year, patients’ ethnicity, study design, source and type of materials, number of cases, tumor stage, histological grade, detection methods and frequency of RARβ2 methylation.

In the case-control studies, the non-cancerous controls were defined as: (1) samples from cancer-free people with or without benign breast disease; (2) normal breast tissue from breast cancer patients. Since it is difficult to unify the definitions of non-cancerous subjects, we combined relevant data from eligible studies on the basis of their original group. In the case-series studies, different subtypes of breast cancer based on tumor stage and/or histological grade were analyzed. According to the American Joint Committee on Cancer (AJCC) staging system [18], stage ≤ II was assigned as early-stage and stage ≥ III was assigned as late-stage. For histological grade, Grade ≤ II was assigned as low-grade and Grade III was assigned as high-grade.

### Statistics analyses

The odds ratios (ORs) with their corresponding 95% confidence intervals (CIs) were used to assess the methylation status of RARβ2 between breast cancer patients and non-cancerous populations, tumor stage and histological grade. The heterogeneity was examined by the Cochran Q test and I^2^ statistic, if acceptable heterogeneity was observed (P≥0.10 and I^2^<40%), a fixed-effect model was used for pooling studies, otherwise, the random-effects model was utilized [19]. Moreover, subgroup analysis was conducted to investigate sources of heterogeneity and differences between ethnicity (Caucasian or Non-Caucasian), sample origin (tissue sample or blood sample) or detection methods (MSP or QMSP). Sensitivity analysis was also performed by omitting any single study at each iteration to assess the stability of the analysis results or by switching the fixed and random effects models. Publication bias was estimated with a visual inspection of funnel plots if the included number of studies was nine or more. All statistical analyses were performed using Review Manager (RevMan) 5.2 software.

## Results

### Study selection and characteristics

**Fig 1:**
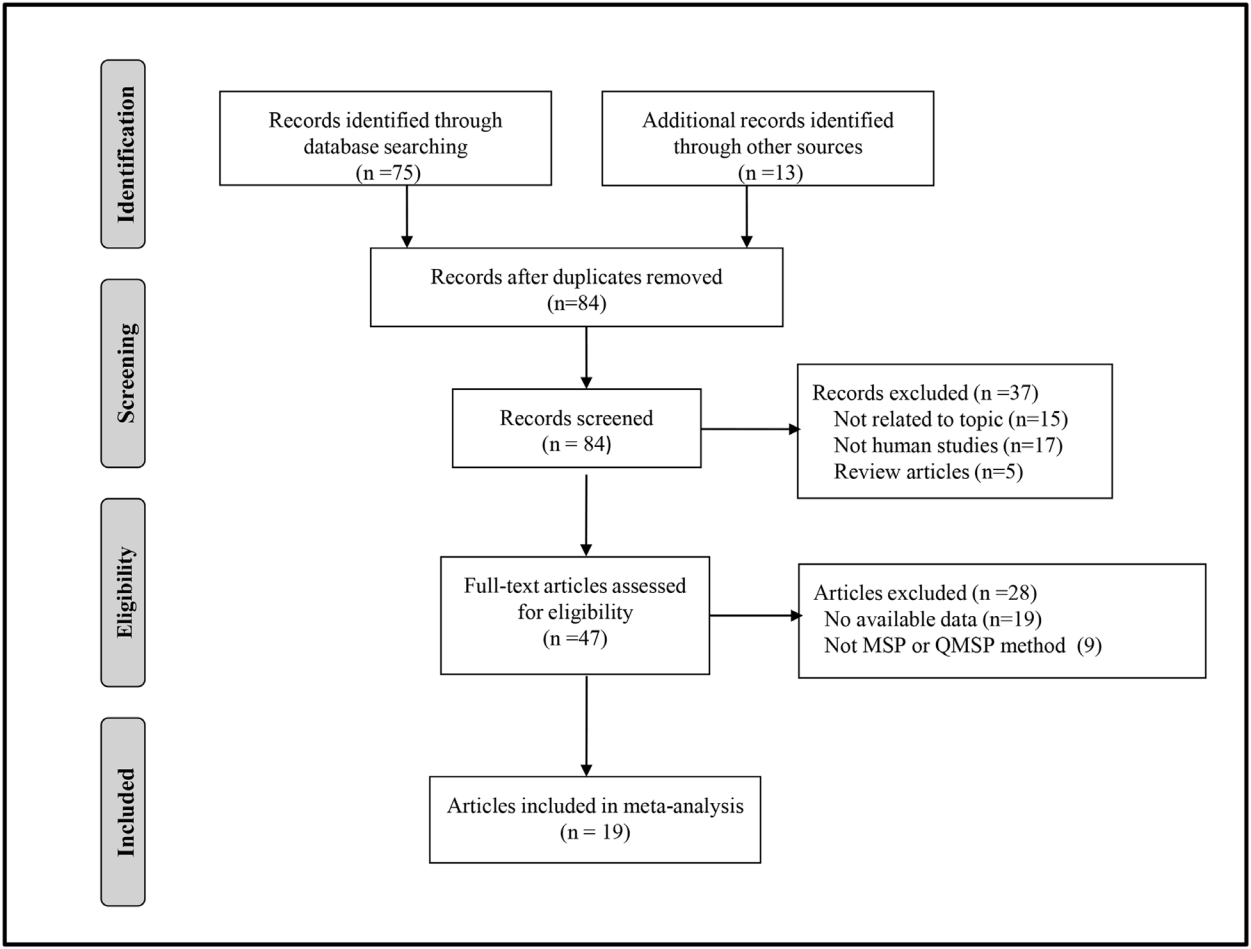
The flow diagram of the study selection process.

We performed a detailed study selection process that is presented in Fig 1 to carefully choose the studies included in our analysis. Since the study by Mirza et al. [20] investigated the methylation status of RARβ2 in both tissue and blood samples of breast cancer patients, we treated the report as two independent studies. Finally, 20 studies [14, 20–37] involving 16 case-control and four case-series studies [24, 28, 32–33] were included in our analysis. Of the included reports, all 16 case-control studies comprising 1,120 cases and 589 controls evaluated the methylation frequency of the RARβ2 promoter in breast cancer and non-cancerous samples. In addition, five case-control and the four case-series studies investigated the association between RARβ2 promoter methylation and tumor stage and histological grade in breast cancer. All of the cases were histologically or pathologically confirmed as breast cancer. The specifics of the studies are summarized in Table 1.

**Table 1:** Characteristics of included studies in the meta-analysis.

						*RARβ2* (M/N)^a^		Stage (M/N)^a^		Grade (M/N)^a^	
References	Ethnicity	Study design	Material	Case sample	Method	Case	Control	Early-stage^b^	Late-stage	Low- grade^c^	High-grade
Evron 2001 [36]	Caucasian	Case-control	Tissue	64	MSP	24/64	0/20	NA	NA	NA	NA
Fackler 2003 [37]	Caucasian	Case-control	Tissue	76	MSP	31/76	0/8	NA	NA	NA	NA
Pu 2003 [14]	Caucasian	Case-control	Tissue	66	MSP	41/66	12/36	NA	NA	NA	NA
Parrella 2004 [21]	Caucasian	Case-control	Tissue	54	MSP	11/54	0/10	NA	NA	NA	NA
Lewis 2005 [22]	Caucasian	Case-control	Tissue	17	MSP	5/17	2/17	NA	NA	NA	NA
Shinozaki 2005 [35]	Caucasian	Case-control	Tissue	151	MSP	36/151	0/10	NA	NA	NA	NA
Hoque 2006 [23]	African	Case-control	Blood	90	QMSP	12/47	3/38	3/24	20/66	NA	NA
Li 2006 [24]	Caucasian	Case-series	Tissue	157	MSP	NA	NA	NA	NA	20/96	22/61
Skvortsova 2006 [25]	Caucasian	Case-control	Blood	20	MSP	3/20	2/25	NA	NA	NA	NA
Bagadi 2008 [26]	Asian	Case-control	Tissue	54	MSP	8/54	0/5	6/27	2/27	6/37	2/14
Jeronimo 2008 [27]	Caucasian	Case-control	Tissue	66	QMSP	35/66	14/43	NA	NA	NA	NA
Tao 2009 [28]	Mixed	Case-series	Tissue	715	QMSP	NA	NA	183/668	18/47	88/322	77/313
Van der Auwera 2009 [29]	Caucasian	Case-control	Tissue	100	QMSP	29/100	0/9	NA	NA	NA	NA
Brooks 2010 [30]	Mixed	Case-control	Blood	45	QMSP	3/45	2/131	NA	NA	NA	NA
Jing 2010 [31]	Asian	Case-control	Blood	50	MSP	21/50	2/50	NA	NA	NA	NA
Karray-Chouayekh 2010 [32]	African	Case-series	Tissue	78	MSP	NA	NA	16/21	18/30	41/61	11/17
Pirouzpanah 2010 [33]	Asian	Case-series	Tissue	96	MSP	NA	NA	NA	NA	15/48	18/48
Mirza 2012 [20]	Asian	Case-control	Tissue-a	100	MSP	23/89	1/15	12/51	14/49	14/50	7/28
Mirza 2012 [20]	Asian	Case-control	Blood-b	100	MSP	20/100	0/30	10/51	10/49	10/50	6/28
Swellam 2015 [34]	African	Case-control	Blood	121	MSP	116/121	11/142	82/86	34/35	85/89	31/32

MSP, methylation-specific PCR, QMSP, quantitative MSP; NA, not available.

^a^ Frequencies of methylated RARβ2;

^b^ Stage ≤ II was assigned as early-stage and stage ≥ III was assigned as late-stage;

^c^ Grade ≤ II was assigned as low-grade and Grade III was assigned as high grade;

### RARβ2 methylation and breast cancer risk

**Fig 2:**
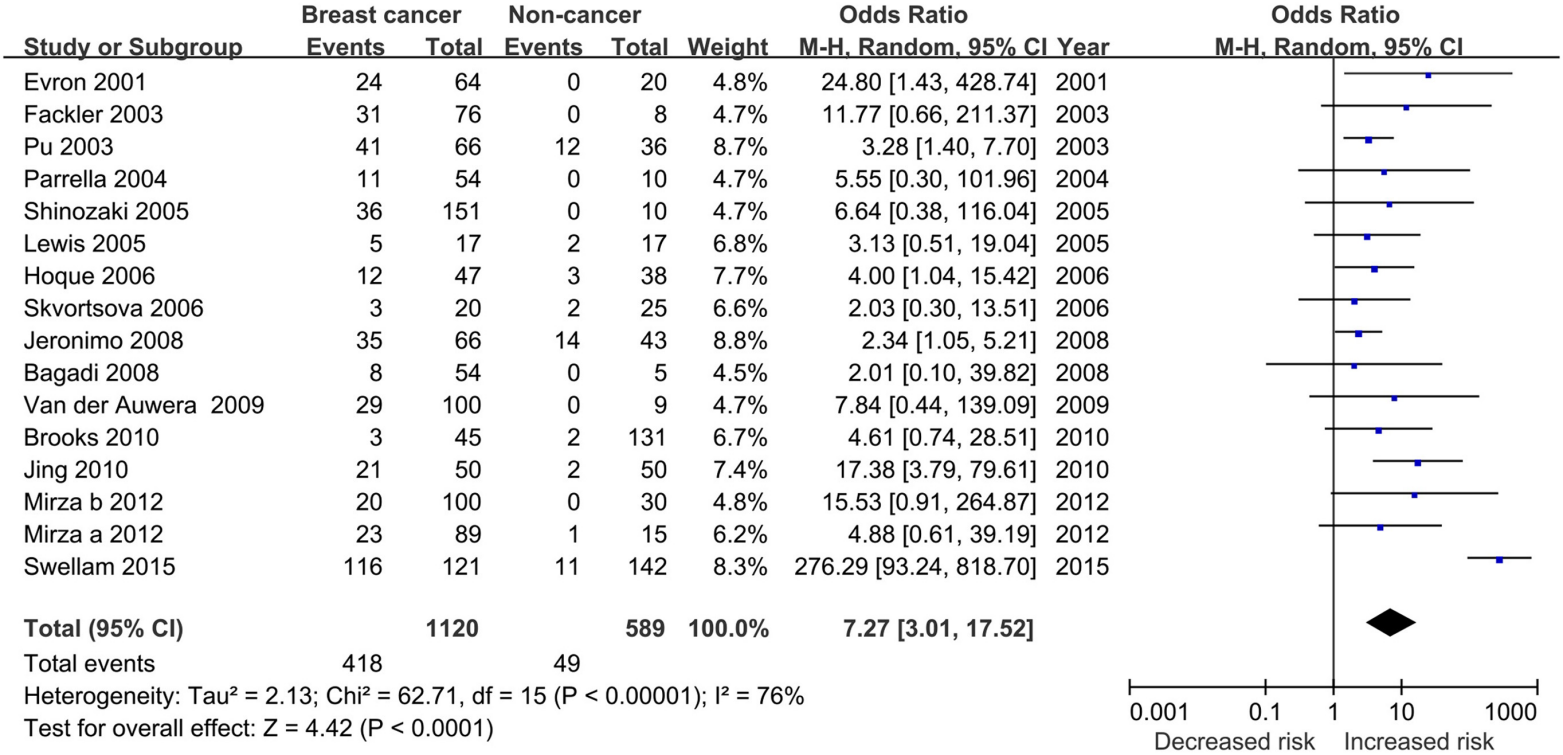
Forest plot of the association between RARβ2 methylation and breast cancer risk based on a random-effects model. The squares and horizontal lines correspond to the OR and 95% CI.

The level of RARβ2 methylation in breast cancer patients was 7.27 times higher than in non-cancerous controls under the random-effects model (OR = 7.27, 95% CI = 3.01–17.52, Fig 2). For this result, subgroup and sensitivity analyses were conducted to investigate the possible source of heterogeneity. Subgroup analysis by ethnicity demonstrated that aberrant methylation of RARβ2 was significantly related to increased breast cancer risk among both Caucasian (OR = 3.88, 95% CI = 2.40–6.26; fixed-effect model) and Non-Caucasian (OR = 13.60, 95% CI = 2.27–81.30; random-effects model) populations. When stratified by material, statistical associations were found between methylation status of RARβ2 and both breast cancer tissue samples (OR = 4.01, 95% CI = 2.49–6.46; fixed-effect model) and blood samples (OR = 12.47, 95% CI = 2.12–73.23; random-effects model). The aberrant methylation of RARβ2 was also statistically associated with breast cancer risk when using MSP (OR = 9.08, 95% CI = 2.85–28.99; random-effects model) and QMSP (OR = 3.15, 95% CI = 1.69–5.88, fixed-effect model) (Table 2).

**Table 2:** Overall and subgroup analyses of RARβ2 methylation and breast cancer risk in case-control studies.

		Test of associations			Test of heterogeneity		
Variables	N	OR (95%CI)	Z	P-value	Model	P*h*	I^2^(%)
Total	16	7.27 (3.01, 17.52)	4.42	<0.0001	R	<0.10	76
**Ethnicity**							
Caucasians	9	3.88 (2.40, 6.26)	5.55	<0.00001	F	0.78	0
Non-Caucasians	6	13.60 (2.27, 81.30)	2.86	0.004	R	<0.10	84
**Material**							
Tissue	10	4.01 (2.49, 6.46)	5.69	<0.00001	F	0.86	0
Blood	6	12.47 (2.12, 73.23)	2.79	0.005	R	<0.10	86
**Method**							
MSP	12	9.08 (2.85, 28.99)	3.73	0.0002	R	<0.10	78
QMSP	4	3.15 (1.69, 5.88)	3.61	0.0003	F	0.75	0

N, number of trials; Non-Caucasians included Asians and Africans; OR, odds ratio; F, fixed-effect model; R, random-effects model.

### Subgroup and sensitivity analyses

In sensitivity analysis, when we removed the study by Swellam et al.[34], the initial heterogeneity (P*h*<0.10, *I* ^2^ = 76%) was reduced to none (P*h* = 0.73, *I* ^2^ = 0%) in evaluating the association of RARβ2 methylation and breast cancer risk (OR = 4.75, 95% CI = 3.18–7.10; fixed-effect model). Moreover, when the data from the heterogeneous study (Swellam et al., 2015) was omitted, the heterogeneity was largely reduced in Non-Caucasian populations (P*h* = 0.53, *I* ^2^ = 0%), blood samples (P*h* = 0.38, *I* ^2^ = 4%) and MSP method (P*h* = 0.65, *I* ^2^ = 0%), without affecting the results (P<0.01). The results were also not significantly changed by switching the effects models. The sensitivity analyses further verified the stability and reliability of our results (Table 3).

**Table 3:** Subgroup analyses of RARβ2 methylation and breast cancer risk by omitting one heterogeneous study (Swellam et al.).

		Test of associations			Test of heterogeneity		
Variables	N	OR (95%CI)	Z	P-value	Model	P*h*	I^2^(%)
Total	15	4.75 (3.18, 7.10)	7.62	<0.00001	F	0.73	0
**Ethnicity**							
Caucasians	9	3.88 (2.40, 6.26)	5.55	<0.00001	F	0.78	0
Non-Caucasians	5	7.53 (3.30, 17.19)	4.8	<0.00001	F	0.53	0
**Material**							
Tissue	10	4.01 (2.49, 6.46)	5.69	<0.00001	F	0.86	0
Blood	5	7.01(3.32, 14.80)	5.1	<0.00001	F	0.38	4
**Method**							
MSP	11	6.04 (3.54, 10.28)	6.62	<0.00001	F	0.65	0
QMSP	4	3.15 (1.69, 5.88)	3.61	0.0003	F	0.75	0

N, number of trials; Non-Caucasians included Asians and Africans; OR, odds ratio; F, fixed-effect model.

### RARβ2 methylation and tumor stage and histological grade

**Fig 3:**
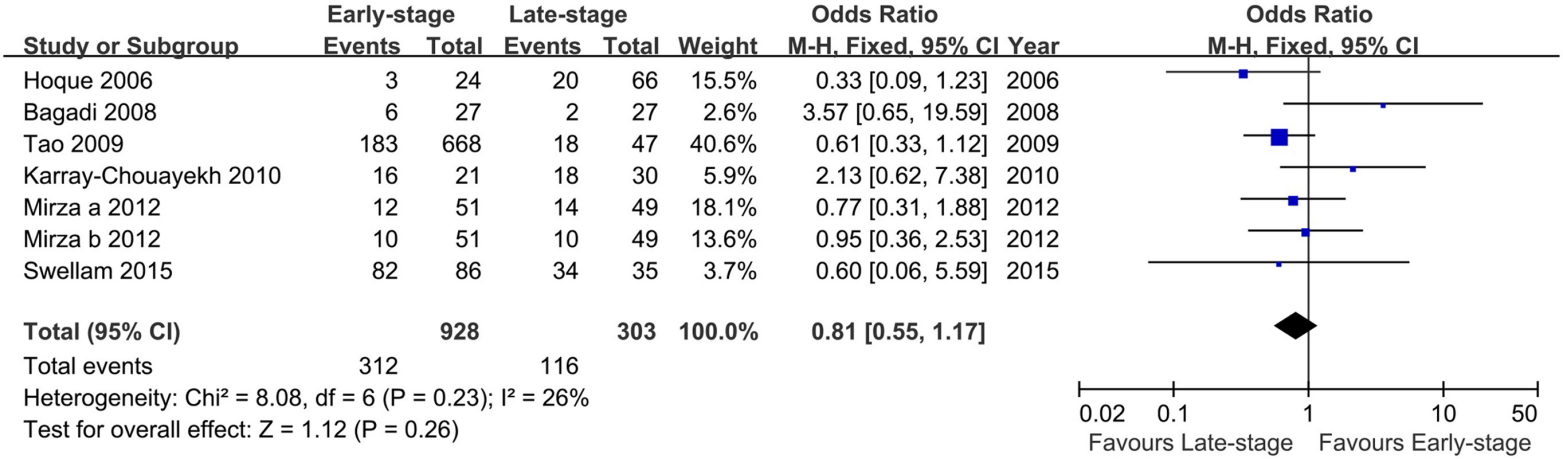
Forest plot of the association between RARβ2 methylation and tumor stage based on a fixed-effect model. The squares and horizontal lines correspond to the OR and 95% CI.

**Fig 4:**
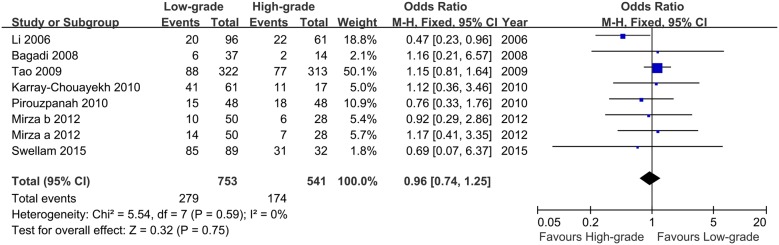
Forest plot of the association between RARβ2 methylation and histological grade based on a fixed-effect model. The squares and horizontal lines correspond to the OR and 95% CI.

A total of seven studies were included in the determination of the OR comparing RARβ2 methylation in early-stage versus late-stage breast cancer under the fixed -effect model. The pooled analysis revealed that no significant relationship existed between methylation status of RARβ2 and breast cancer stage (OR = 0.81, 95% CI = 0.55–1.17, Fig 3). The correlation of RARβ2 methylation and histological grade was also compared using the fixed-effect model. The pooled OR from the eight included studies showed that no association was observed between the RARβ2 methylation status of low-grade breast cancer samples compared to high-grade samples (OR = 0.96, 95% CI = 0.74–1.25, Fig 4).

### Publication bias

**Fig 5:**
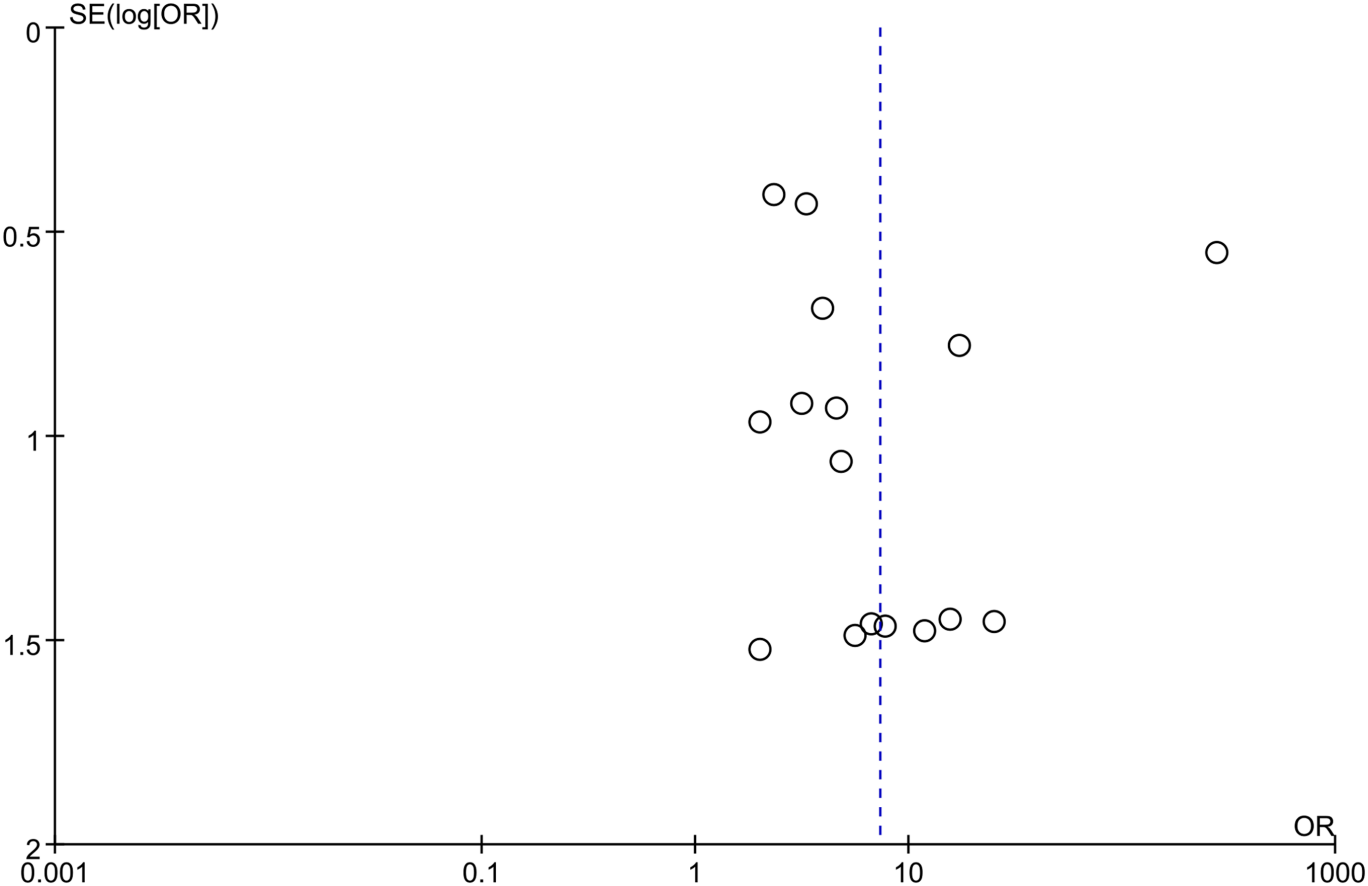
Funnel plot for evaluating publication bias for RARβ2 methylation and breast cancer risk. The standard error of log (OR) of each study was plotted against its log (OR).

The funnel plot appeared asymmetrical in the assessment of RARβ2 methylation status in breast cancer samples compared to non-cancerous controls (Fig 5), indicating publication bias may exist. Since only a limited number of studies were included in the assessments of RARβ2 methylation status and tumor stage and histological grade, publication bias was not tested.

## Discussion

### Main findings

The results of our meta-analysis indicate that aberrant methylation of RARβ2 is more frequently observed in breast cancer than in non-cancerous controls. Hence, we carried out this meta-analysis [38–41]. These results were found when comparing either tissue or blood samples, among both Caucasian and Non-Caucasian populations and by MSP or QMSP methods. We did not observe any significant associations between RARβ2 methylation status and breast cancer stage or histological grade.

Several studies have found that breast tumors exhibit a higher frequency of RARβ2 methylation than non-cancerous counterparts [14, 27, 31]. In this meta-analysis, we analyzed 16 reports comprising 1,120 cases and 589 controls to further confirm the status of RARβ2 methylation in breast cancer versus controls. We found that the methylation frequency of RARβ2 in breast cancer was 7.27 times greater than that in non-cancerous subjects, indicating that RARβ2 could serve as a potential risk factor in breast cancer detection. It is well known that the incidence rates and distribution patterns of breast cancer are different among patients of various ethnic groups [42]. Our analysis demonstrated that the detection of RARβ2 methylation has significant implications in both Caucasian and Non-Caucasian populations, suggesting that RARβ2 methylation status may be able to be utilized as a novel molecular biomarker. Moreover, the detection of RARβ2 methylation in blood samples would be useful as a non-invasive diagnostic tool in breast cancer screening. MSP (non-quantitative) and QMSP are two commonly utilized sodium bisulfite treatment-based detection assays to examine gene methylation. According to our results, these two techniques are similarly effective in deciphering RARβ2 methylation in breast cancer samples compared to non-cancerous controls.

Previously, Hoque et al. [23] demonstrated that tumors with frequent methylation of RARβ2 were more often detected in late-stage compared to early-stage breast cancer. Moreover, a statistical inverse association between histological grade and RARβ2 hypermethylation was reported in two studies [24, 28]. On the contrary, other studies have suggested that no significant associations exist between RARβ2 methylation and tumor stage or histological grade [20, 26, 32]. The current meta-analysis confirmed that no apparent associations exist between the methylation distributions of RARβ2 and tumor stage or histological grade, indicating that the promoter methylation of RARβ2 may be an early molecular event in breast cancer development.

### Potential biological mechanism

Breast cancer is considered to be a multifactorial and hormone dependent disease, arising from the activation of oncogenes and silencing of tumor suppressor genes [24]. It has been demonstrated that epigenetic aberrancies known to occur in breast cancer play an important role in the inactivation of functionally important tumor suppressors. In breast cancer, several critical genes reportedly undergo aberrant hypermethylation, including genes involved in cell cycle regulation (p16, Cyclin D2), cell apoptosis (DAPK), DNA repair (BRCA1), cell adhesion (CDH1) and cell signal transduction (ER and RARβ2) [24, 26]. Hypermethylation of CpG-rich areas in gene promoters is correlated with chromatin condensation, replication delay, transcriptional inhibition and gene silencing [28]. As previously reported, RARβ2 is a tumor suppressor gene, and loss of expression of RARβ2 due to aberrant methylation status is observed during breast carcinogenesis [20, 43]. Additionally, the RARβ2 gene is known to be induced by retinoic acid, which possesses anti-proliferative and apoptosis-inducing properties, suggesting that inactivation of the RARβ2 gene expression may provide a local cellular environment favorable for tumor progression [10].

In addition to DNA methylation, RARβ2 transcription can also be regulated by histone modifications. Deacetylation and acetylation on lysine residues of histone amino-terminal tails play important roles in gene transcription. The RARβ2 promoter, containing several high-affinity RA-responsive elements (RAREs), is normally mediated by a dynamic histone deacetylase (HDAC) and histone acetyltransferase (HAT) balance in the presence of physiological levels of RA. However, increased level of histone deacetylation was observed during epithelial cell tumorigenesis and appropriate level of histone reacetylation at RARβ P2 can lead to reactivation of endogenous RARβ2 transcription [44]. On the other hand, Wang et al. has revealed significant inverse association between RARβ2 promoter methylation and its gene expression (r = -0.322; p<0.05), suggesting that RARβ2 transcriptional silencing is at least partly caused by DNA methylation at RARβ2 promoter [45].

Studies demonstrated that impaired integration of RA signal via the RA receptor α (RARα), can lead to RARβ2 epigenetic silencing, which is marked by the repressed chromatin status of RARβ2, including DNA hypermethylation [46–47]. In breast cancer cells, several proteins involved in RA transport and/or metabolism were found to be deranged. There is evidence that mutations in the cellular RA-binding protein 2 (CRABP2), which channels RA onto nuclear RARα can trigger the deranged CRABP2 function and result in epigenetic repression of the RARα direct target RARβ2 [48]. Recently, preferentially expressed antigen in melanoma (PRAME) has been described as a tumor antigen and is overexpressed in a variety of cancers. PRAME is located at the RAR target promoters and served as a dominant repressor of RA signaling through interacting with RARα; thus, aberrant expression of RARα and PRAME can inhibit RA-induced growth arrest and apoptosis [49].

### Strengths and limitations

A few limitations of this meta-analysis should be considered. First, the lack of sufficient data provided in reports restricted further evaluation of potential associations between the *RARβ2* methylation and other confounding factors, such as age, hormone receptor status and subtype of breast cancer, which might be sources of the heterogeneity. Second, certain heterogeneity existed between the included studies, which may reflect differences in patients’ ethnicity, material type, detection methods and definition of the control groups. Third, publication bias existed, potentially because only published studies written in English or Chinese were identified as eligible studies. Additionally, publication bias for the analyses comparing *RARβ2* methylation and breast cancer stage and grade was not assessed due to the limited number of included studies.

Although this report does have some limitations, this study contains a number of strengths. Most importantly, this is the first meta-analysis conducted to investigate the association between *RARβ2* methylation and breast cancer risk. We identified relevant published reports through a systematic search strategy, aiming to collect all eligible studies that met the inclusion criteria to ensure that our analysis was reliable and scientific. In addition, subgroup analysis was performed and determined that *RARβ2* methylation associated with breast cancer risk according to patients’ ethnicity, type of material tested and detection method utilized, thus indicating the robustness of our findings. Furthermore, the relationship of *RARβ2* methylation with breast cancer risk remained significant in the sensitivity analysis when different methodologies were used.

## Conclusion

In summary, our results reveal that aberrant *RARβ2* promoter methylation may contribute to breast cancer susceptibility. The detection of *RARβ2* methylation could offer an alternative approach for early non-invasive diagnosis and monitoring of breast cancer. However, it must be taken into consideration that DNA methylation is only a component of the observed gene inactivity, and RARβ2 methylation may underestimate RARβ2 transcriptional silencing. Thus, well-designed clinical trials with larger sample sizes are needed in future studies.

## Supporting Information

S1 PRISMA Checklist The PRISMA Checklist of this meta-analysis. (DOC)

S1 Table The primer sequences used in the selected studies of RARβ2 promoter methylation in breast cancer. (XLSX)

S2 Table The list of full-text excluded articles. (XLSX)
